# Reference cells and ploidy in the comet assay

**DOI:** 10.3389/fgene.2015.00061

**Published:** 2015-02-27

**Authors:** Gunnar Brunborg, Andrew Collins, Anne Graupner, Kristine B. Gutzkow, Ann-Karin Olsen

**Affiliations:** ^1^Department of Chemicals and Radiation, Division of Environmental Medicine, Norwegian Institute of Public Health, OsloNorway; ^2^Department of Nutrition, University of Oslo, OsloNorway

**Keywords:** comet assay, genome size, testicular cells, fish cells, reference cells

## Abstract

In the comet assay single cells are analyzed with respect to their level of DNA damage. Discrimination of the individual cell or cell type based on DNA content, with concomitant scoring of the DNA damage, is useful since this may allow analysis of mixtures of cells. Different cells can then be characterized based on their ploidy, cell cycle stage, or genome size. We here describe two applications of such a cell type-specific comet assay: (i) Testicular cell suspensions, analyzed on the basis of their ploidy during spermatogenesis; and (ii) reference cells in the form of fish erythrocytes which can be included as internal standards to correct for inter-assay variations. With standard fluorochromes used in the comet assay, the total staining signal from each cell – whether damaged or undamaged – was found to be associated with the cell’s DNA content. Analysis of the fluorescence intensity of single cells is straightforward since these data are available in scoring systems based on image analysis. The analysis of testicular cell suspensions provides information on cell type specific composition, susceptibility to genotoxicants, and DNA repair. Internal reference cells, either untreated or carrying defined numbers of lesions induced by ionizing radiation, are useful for investigation of experimental factors that can cause variation in comet assay results, and for routine inclusion in experiments to facilitate standardization of methods, and comparison of comet assay data obtained in different experiments or in different laboratories. They can also be used – in combination with a reference curve – to quantify the DNA lesions induced by a certain treatment. Fish cells of a range of genome sizes, both greater and smaller than human, are suitable for this purpose, and they are inexpensive.

## INTRODUCTION

In our past studies of genotoxicity in mixtures of primary cultures of testicular cells from rats and humans (Bjorge et al., 1996a), there was a need to characterize subpopulations of spermatogenic cells. Flow cytometric analysis of DNA-stained cells was used for this purpose. We applied alkaline filter elution to measure DNA damage in testicular cell populations partly purified by means of centrifugal elutriation. We subsequently found that different testicular cell types could be identified when using the comet assay, which – unlike alkaline elution – allows measurement of DNA damage in individual cells (Bjorge et al., 1996b). This was possible since the fluorescent signal in the comet assay is related to the amount of DNA, which depends on cell ploidy. Testicular cell suspensions contain spermatogonia and secondary spermatocytes (but also Sertoli and Leydig somatic cells; 2n), primary spermatocytes (4c), secondary spermatocytes after first meiotic cleavage (2C), and spermatids and spermatozoa at different stages of differentiation and maturation (1n). The relative proportions of these cell types are specific for human and rat testicular cells (Bjorge et al., 1996a; Olsen et al., 2003). Provided that scoring conditions (light intensity and staining) are standardized, the response of cell populations may be compared in different comet assay experiments. We used these approaches to estimate DNA damage induction and its repair in spermatogenic cells from mixed testicular cell populations (Olsen et al., 2001, 2003).

Cell-specific fluorescence can also be used as a basis for reference cells in the comet assay, as will be shown here. Reference cells are useful in standardization of conditions during various stages of the experimental protocol. Unexpected variations in measurement of specific DNA lesions occur between laboratories (Forchhammer et al., 2010), even for experienced comet assay users. Calibration trials, validation efforts, standardization of methods, and comparison of comet results between experiments and laboratories should profit from reference cells which could be analyzed in parallel with the sample cells. Reference cells could be in neighboring gel samples, or – preferably – mixed with the samples cells before the comet analysis and therefore subjected to exactly the same treatment conditions at all steps of the comet assay protocol (the only requirement being that the reference comets should be distinguishable from the comets from sample cells). This should also allow a better control of local variations in electrophoresis conditions. Zainol et al. (2009) developed a comet assay using internal bromodeoxyuridine-prelabelled reference cells that were identified on the basis of *in situ* immunostaining before scoring. Such cells can be mixed with unlabelled cells. This very useful approach, however, involves extra treatment steps and – compared with a method based on differential DNA content – is more costly because the method relies on antibodies.

We describe how DNA content-specific fluorescence intensity in the comet assay can be used, (i) for characterization of testicular cell populations; and (ii) as a convenient and low-cost system for internal reference cells taking advantage of the lower DNA content of some species of fish.

## MATERIALS AND METHODS

Human testicular biopsies were obtained from organ donors, and single-cell suspensions of testicular cells were prepared as described (Bjorge et al., 1996a). Cells (unfrozen) were processed for comet assay analysis, stained with ethidium bromide, and analyzed using the Fenestra Comet image analysis system (Kinetic Imaging LTD, Liverpool, UK; Bjorge et al., 1996b). Comet Tail Moments (TM; these experiments were partly done in the early days of the comet assay, when TM was often used) and total fluorescence intensity (TFI) were recorded for each cell; these parameters are already integrated in Fenestra. During scoring no cells were excluded on the basis of either a very strong or a very weak fluorescence signal.

Blood (1–2 mL) was drawn using a 2 mL syringe from the tail vein of a turbot (*Scophthalmus maximus*) weighing about 600 g, kept in an indoor aquarium (about 50 m^3^, 8°C, continuously flushed with fresh sea water from the Oslo fjord at Solbergstrand, Drøbak, 40 km south of Oslo). The blood was diluted 1:10 in ice-cold phosphate buffered saline (PBS; 10 mM PO_4_^3-^, 137 mM NaCl, and 2.7 mM KCl) with 10 mM ethylenediaminetetraacetic acid (EDTA), pH 7.4; or in RPMI1640 medium (w/Hepes and glutamine; pH 7.4); and transported on ice to the laboratory in Oslo. After microscopic examination and counting, samples of the fish erythrocytes (FE), which are nucleated, were diluted once more 1:10 in RPMI1640 but now containing 10% dimethyl sulfoxide (DMSO) and 20% foetal calf serum (FCS), and 1 mL aliquots were frozen slowly to -80°C while placed in a Mister Frosty^TM^ Freezing Container (Thermo Scientific, Oslo, Norway) unit containing isopropanol, for slow freezing of biological samples (the temperature is reduced at a rate of approximately 1° per minute). For use, samples were thawed by warming for a few seconds to allow the frozen ice to be transferred into a tube containing 10 mL RPMI medium followed by centrifugation for 10 min at 400 ×*g* and washing in the same medium. Suitable aliquots were mixed 1:10 in LMP agarose (0.7%) and analyzed according to our protocol for 96 minigels and electrophoresis with circulation (Gutzkow et al., 2013). Fresh (unfrozen) samples were diluted in RPMI medium (without DMSO/serum), mixed with agarose, and analyzed in the same way. The FE were added to GelBond^®^ films either as independent samples, or they were mixed 1:1 with human peripheral blood mononuclear cells (PBMN), which had been previously frozen, before embedding in agarose on GelBond^®^ films (for details, see Gutzkow et al., 2013). Photomicrographs of stained cell samples were recorded using Comet IV (see below). In some experiments, samples were irradiated before analysis with defined doses of X-rays (260 KeV, filtered through 0.5 mm Cu, dose rate 10 G/min) on ice.

For scoring of FE and PBMN comets, dried GelBond films^®^ were rehydrated and stained with SYBRGold (Life Technologies Ltd, Paisley, UK; 2 μL in 25 mL TE buffer pH 7.4, 20 min at RT), rinsed in water, and analyzed with Comet IV (Perceptive Instruments Ltd, Bury St. Edmunds, UK). An Olympus BX51 fluorescence microscope with CCD camera was used. Tail%DNA (TD) and TFI were recorded for each cell; these parameters are already integrated in Comet IV. During scoring no cells were excluded on the basis of either a very strong or a very weak fluorescence signal.

Statistics: Means and frequency distributions were calculated using Microsoft Excel.

## RESULTS

### COMET ANALYSIS OF TESTICULAR CELL SUSPENSIONS

**FIGURE 1:**
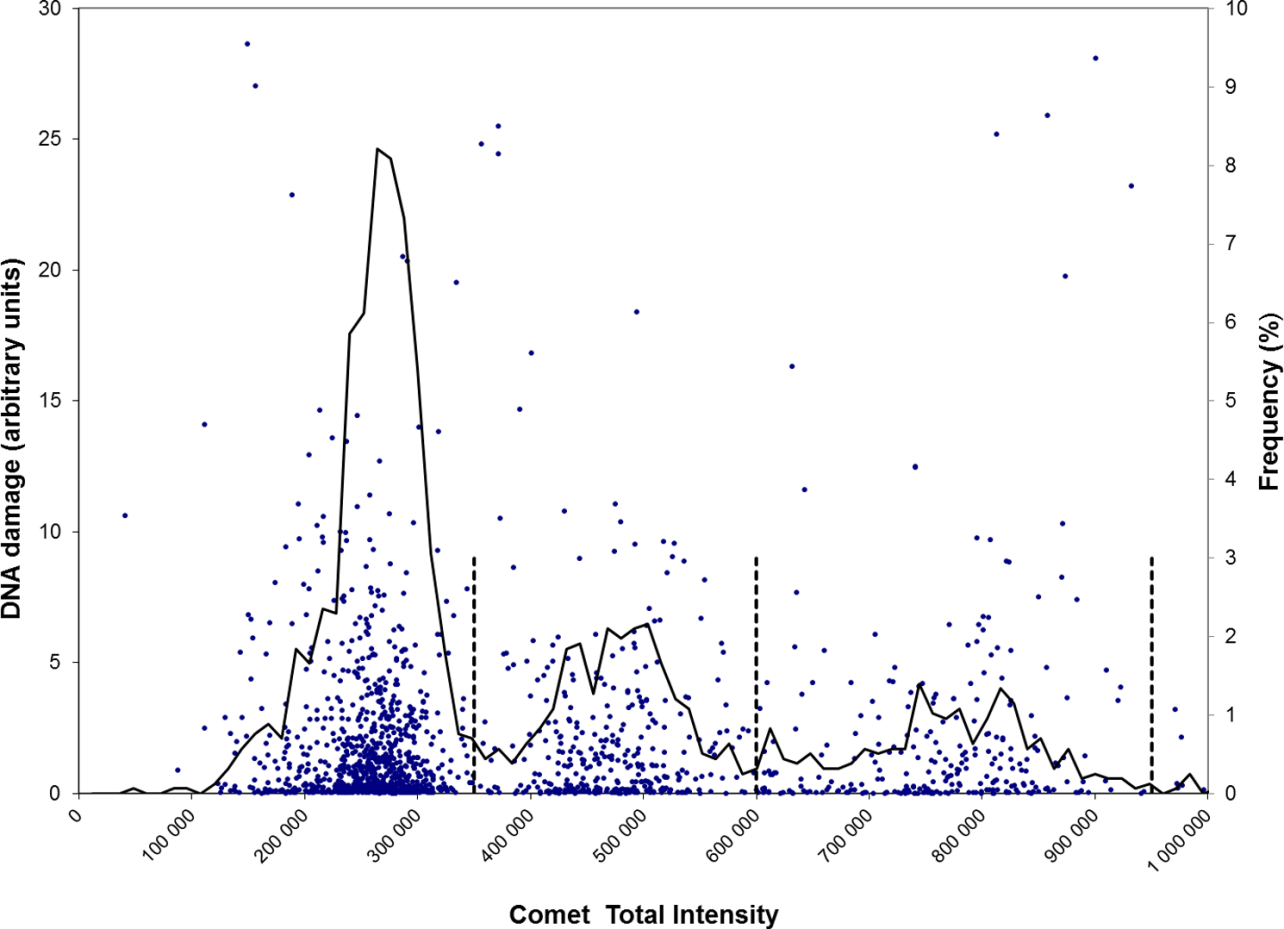
**Scatter plots of DNA damage of cells of different ploidy.** Each single cell (with varying tail size) is scored for DNA damage together with its total fluorescence intensity (TFI). DNA damage (left vertical axis) and TFI (horizontal axis) were determined for 1600 control cells (dots) prepared from a human testicular biopsy. The stippled vertical lines indicate the partitions of three different populations of cells, of ploidy 1n, 2n, and 4c (discriminated at fluorescence intensity 350 000, 600 000, and 950 000, respectively). The mean intensities of cells within these classes are 255 000, 471 000, and 761 000 for 1n, 2n, and 4c, respectively. Mean levels of DNA damage (TM) are in the range 2.2–2.5 for the three classes and are not significantly different. Plotted line: mean frequency (%, right vertical axis) of DNA damage at each intensity level.

The results shown in **Figure 1** were obtained by pooling all data from an analysis of 16 samples of unexposed (control) fresh human testicular cells (from one donor). The data were sorted according to TFI values and they fell into three distinct classes. Based on the distribution of the total intensities, thresholds were sought for separation of the three populations of cells (vertical lines in **Figure 1**). The profile resembles the graphs obtained with flow cytometric analysis of testicular cells (Bjorge et al., 1996b). The mean fluorescence intensities (≈ DNA content) are given in the legend. These cells were not subjected to genotoxic treatment and the levels of DNA damage in the three classes were low and very similar.

### REFERENCE CELLS FROM FISH WITH LOW GENOME SIZE

In initial experiments, we tried fish cells from various genera. The polar cod, *Boreogadus saida*, has a suitable genome size (0.88 pg haploid genome; http://www.genomesize.com/), but the intrinsic level of DNA damage in frozen cells was high and variable. Rainbow trout (*Oncorhynchus mykiss*) had less DNA damage but its genome size (2.4–2.7 pg) is quite similar to that of *Homo sapiens* (3.50 pg); this was obvious in scatter plots similar to **Figure 3** (see below) but without the two distinct populations (data not shown). In the subsequent experiments we used the turbot (*Scophthalmus maximus;* 0.86 pg).

**FIGURE 2:**
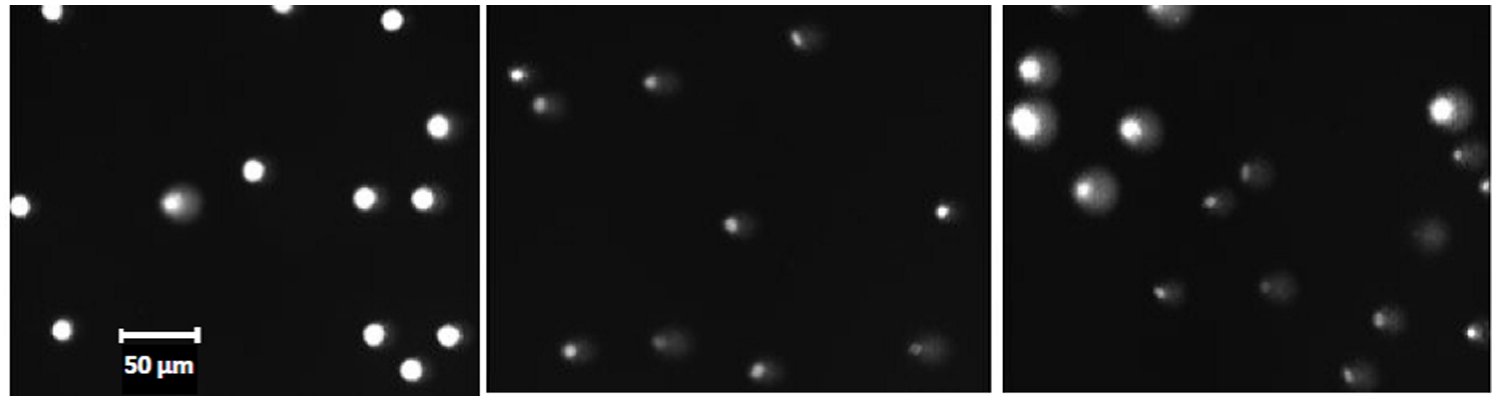
**Micrographs of control (unirradiated) erythrocytes from the turbot fish and human blood mononuclear cells.** Left: human lymphocytes; center: fish erythrocytes (FE); right:1:1 mixture of the two cell types. Representative microscopic fields are shown. Size bar: 50 μm for all micrographs. See Materials and Methods for further details.

**FIGURE 3:**
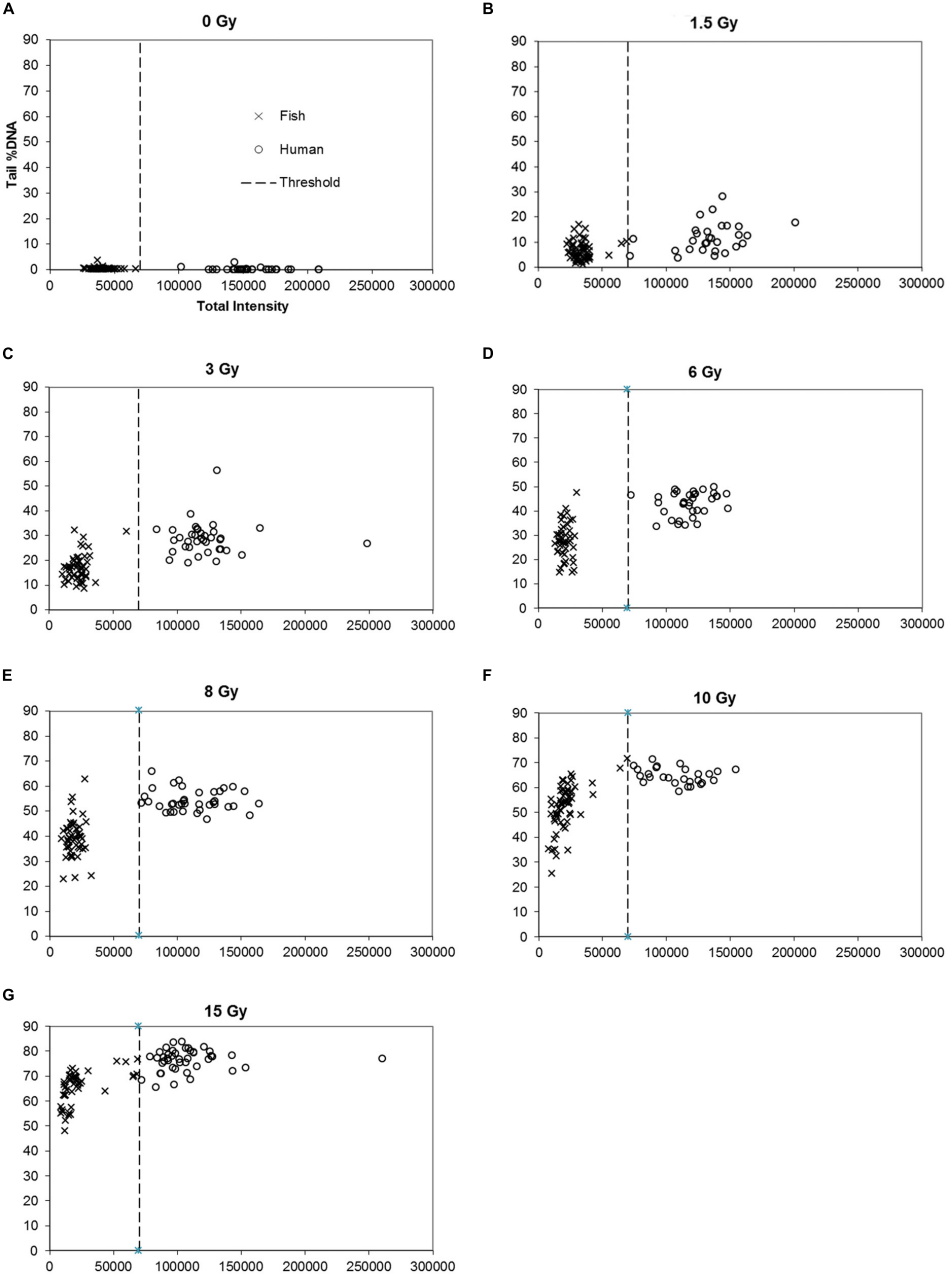
**(A–G)** Mixtures of fish erythrocytes and human PBMN cells exposed to ionizing radiation. Scatter plots are shown for DNA damage (Tail %DNA, y-axis) vs. Total Fluorescence Intensity (arbitrary units, x-axis). X-irradiation doses are indicated; x- and y-axis and symbols are equal for **A–G**.

**Figure 2** illustrates the appearance in fluorescence microscopy of the different control (unexposed) cells of HPBL and FE, embedded either in separate gels or mixed 1:1 in the same gel before analysis with the comet assay. As with the testicular cells, the mixed cell population of control cells (HPBL and FE) belong to distinct classes but in this case there are only two classes (**Figure 3A**).

**FIGURE 4:**
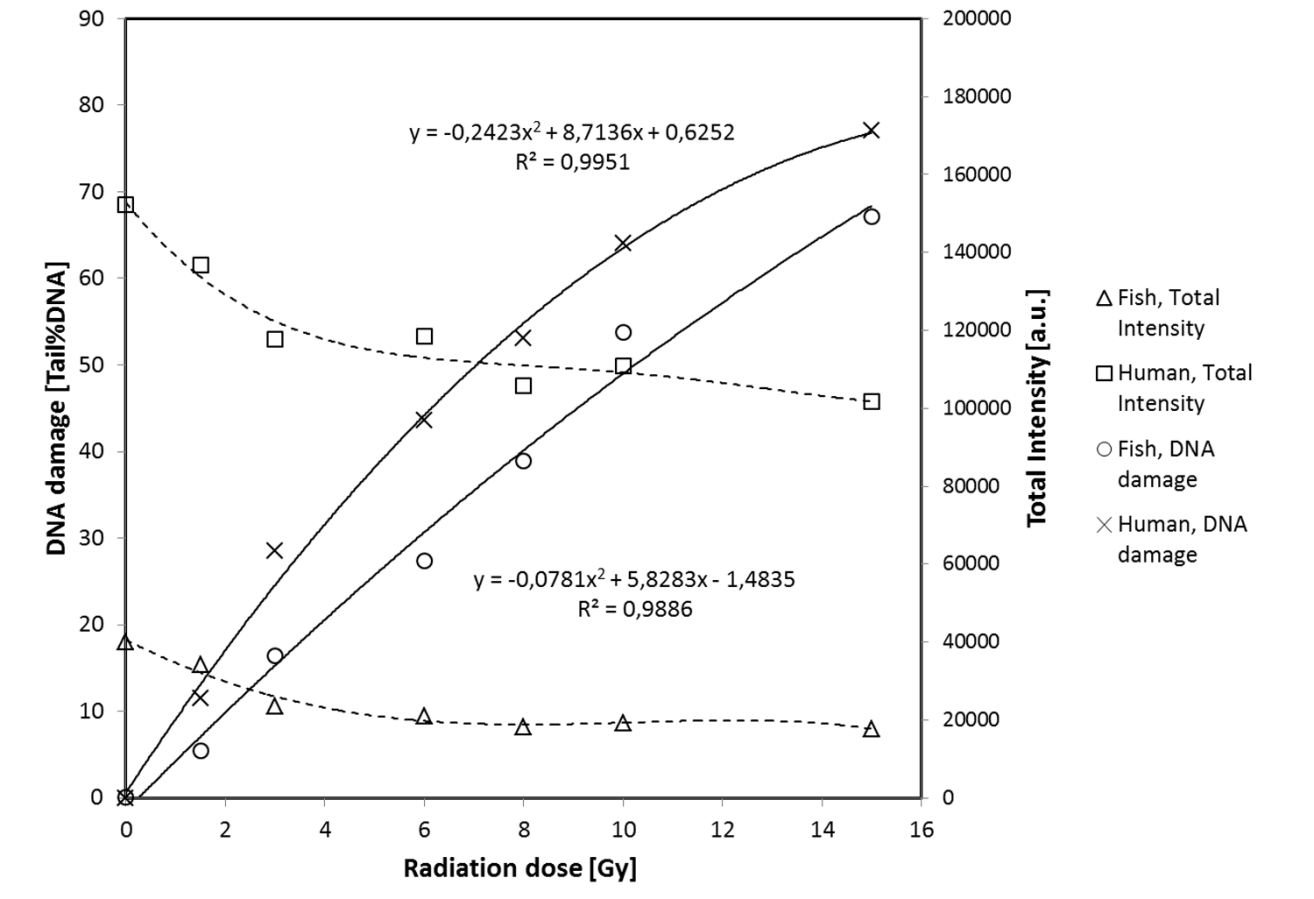
**Fish erythrocytes and human PBMN cells exposed to ionizing radiation.** Left y-axis, medium level of DNA damage (Tail %DNA) vs. X-ray dose (x-axis). Right y-axis, median Total Fluorescence Insensity (arbitrary unit) vs. X-ray dose. Data are derived from **Figure 3**.

The mean TFI for turbot FE in **Figure 3A** is approximately 3–4 times lower than for HPBL, as expected from the genome size differences (see also **Figure 4**). It is apparent from the figure that there are no cells with TFI between 70,000 and 100,000. The absolute threshold value is subject to inter-experimental variations depending on the intensity of the lamp and the staining of DNA. Traditional fluorescent light sources (Mercury Xenon) produce less light with time of use, but newer technologies (liquid light guide combined with metal halide or LED light) solve this latter problem.

Other FE and HPBL samples were exposed to of X-rays (0, 1.5, 3, 6, 8, 10, or 15 Gy) on ice and analyzed quickly to prevent repair of DNA strand breaks. The graphs in **Figure 3** show scatter plots of DNA damage (TD) of the cell populations. The TD values may be discriminated and analyzed separately for the two populations (i.e., intensity levels either below 70,000 or above 100,000). Mean TD values were calculated for each population in **Figure 3** and used to construct the dose response curves for both cell types presented in **Figure 4**. DNA damage increases linearly with the radiation dose for both fish and human cells, except at the highest level of damage (TD > 80%) induced by 15 Gy which is beyond the dynamic range of the assay. In contrast to the DNA damage, the mean TFI does not change much with radiation dose. The slopes of the two dose-response curves are different, i.e., the comet assay indicates that less DNA damage is induced per unit radiation dose in fish than in human cells. This may be related to the lower DNA content of turbot cells, implying a smaller target size for ionizing radiation.

## DISCUSSION

With the testicular cell suspensions, the comets giving strongest fluorescence represent 4c cells (i.e., premeiotic spermatocytes). This is a relatively small population (17.2% of total), compared to the larger proportion of 59.4% 1n cells (post-meiotic haploid spermatids) and 23.5% 2n (2C secondary spermatocytes plus 2n Leydig/Sertoli somatic cells); these data are all derived from **Figure 1**. In a normal experiment, the standard deviation of the small population (low numbers) of 4c cells is higher than for the other populations. We have in some cases selectively scored strongly fluorescing cells, in order to measure DNA damage in this class of 4c cells with higher precision. With automated imaging (e.g., IMSTAR^TM^ Pathfinder, Paris, France), larger numbers of cells may be scored. To estimate the ratio of 2C spermatocytes vs. 2n somatic cells, flow cytometric analysis of vimentin-stained cells may be used.

Fish erythrocytes from the turbot fish have significantly lower genome size than human cells, but there is sufficient DNA in the fish cell to produce a good fluorescent signal (**Figure 2**). Unlike the polar cod, fresh samples of the turbot showed very low background levels of DNA damage. However, turbot FE that had been frozen and stored for a few weeks at -80°C before thawing and analysis, expressed significantly elevated levels of DNA damage which also increased with longer periods of storage (data not shown). Further optimization should be performed to improve the integrity of turbot cell DNA (either FE or other cell types) upon storage. It is well known that human lymphocytes can keep their DNA integrity during freezing and storage for months, both as control and irradiated samples. The ultimate aim would be to prepare and distribute frozen samples of fish cells, either untreated or treated with defined doses of ionizing radiation inducing known levels of DNA damage. Such cells can be used as standards for calculation of frequencies of induction of DNA lesions in sample cells treated with genotoxicants. As an alternative to frozen cells, fresh blood samples may be obtained cheaply and reproducibly from fish living in an aquarium and the same fish may be sampled many times. Collection of blood from the tail vein is not known to harm the fish or to induce disease or pathological changes. The trivial fact that the turbot is a flatfish contributes to easy handling and blood collection, even for untrained personnel. One milliliter of peripheral blood contains sufficient numbers of erythrocytes for 100s of experiments.

The fluorochrome used in these analyses does not seem to be crucial, since we obtained consistent results with both ethidium bromide and SYBRGold. However, care should be taken to avoid saturation of the light signal, since the quantitative relationship with DNA content would then be distorted.

Cells of different ploidy, or in different stages of mitosis and meiosis, may be analyzed using the methodologies described here, and this could represent a cheap (although much more time-consuming) alternative to staining and analysis of the cell cycle distribution of a cell population, or of mixed cell populations, by flow cytometry. Furthermore, very few (as low as 100) cells are needed. Kruszewski et al. (2012) recently used the same methodology to show that the fluorescence intensity corresponds to the position in the cell cycle of dividing cultures. The effect of the cell cycle on induction and repair of DNA damage may this be analyzed. In conclusion, the methods described here represent new applications of the comet assay. Some further validation should be useful.

## Conflict of Interest Statement

The authors declare that the research was conducted in the absence of any commercial or financial relationships that could be construed as a potential conflict of interest.
