# Corrigendum: Reference cells and ploidy in the comet assay

**DOI:** 10.3389/fgene.2017.00004

**Published:** 2017-01-24

**Authors:** Gunnar Brunborg, Andrew Collins, Anne Graupner, Kristine B. Gutzkow, Ann-Karin Olsen

**Affiliations:** ^1^Division of Environmental Medicine, Department of Chemicals and Radiation, Norwegian Institute of Public HealthOslo, Norway; ^2^Department of Nutrition, University of OsloOslo, Norway

**Keywords:** comet assay, genome size, testicular cells, fish cells, reference cells

There is an error in the Acknowledgments, as published, as funding from Research Council of Norway, through its Centres of Excellence funding scheme, was omitted. The correct Acknowledgment appears below. The authors apologize for this error, which does not affect the scientific conclusions of the article in any way.

## Conflict of interest statement

The authors declare that the research was conducted in the absence of any commercial or financial relationships that could be construed as a potential conflict of interest.

